# Protease inhibitor 15, a candidate gene for abdominal aortic internal elastic lamina ruptures in the rat

**DOI:** 10.1152/physiolgenomics.00004.2014

**Published:** 2014-05-01

**Authors:** Samreen Falak, Sebastian Schafer, Amelie Baud, Oliver Hummel, Herbert Schulz, Dominique Gauguier, Norbert Hubner, Mary Osborne-Pellegrin

**Affiliations:** ^1^Max Delbrück Center for Molecular Medicine, Berlin, Germany;; ^2^Wellcome Trust Centre for Human Genetics, University of Oxford, Oxford, United Kingdom;; ^3^Institute of Cardiometabolism and Nutrition, University Pierre & Marie Curie, Hospital Pitié Salpetrière, Paris, France; and; ^4^Inserm U698-University Paris 7, Hôpital Bichat, Paris, France

**Keywords:** subcongenics, heterogeneous stock, rupture of the internal elastic lamina, protease inhibitor 15

## Abstract

The inbred Brown Norway (BN) rat develops spontaneous ruptures of the internal elastic lamina (RIEL) of the abdominal aorta (AA) and iliac arteries. Prior studies with crosses of the BN/Orl RJ (susceptible) and LOU/M (resistant) showed the presence of a significant QTL on chromosome 5 and the production of congenic rats proved the involvement of this locus. In this study, we further dissected the above-mentioned QTL by creating a new panel of LOU.BN(chr5) congenic and subcongenic lines and reduced the locus to 5.2 Mb. Then we studied 1,002 heterogeneous stock (HS) rats, whose phenotyping revealed a low prevalence and high variability for RIEL. High-resolution mapping in the HS panel detected the major locus on chromosome 5 (log *P* > 35) and refined it to 1.4 Mb. Subsequently, RNA-seq analysis on AA of BN, congenics, and LOU revealed expression differences for only protease inhibitor 15 (Pi15) gene and a putative long intergenic noncoding RNA (lincRNA) within the linkage region. The high abundance of lincRNA with respect to reduced Pi15 expression, in conjunction with exertion of longitudinal strain, may be related to RIEL, indicating the potential importance of proteases in biological processes related to defective aortic internal elastic lamina structure. Similar mechanisms may be involved in aneurysm initiation in the human AA.

the inbred brown norway (BN) rat develops spontaneous ruptures of the internal elastic lamina (RIEL) in the abdominal aorta (AA) and iliac arteries (IAs) but not in the thoracic aorta (TA) and also presents an aortic elastin deficiency (4, 7, 19, 32). The RIEL are associated at their early stage with damage to the endothelium and underlying smooth muscle cells causing platelet adhesion followed by rapid cellular repair (7, 30), but the gap in the internal elastic lamina (IEL) remains throughout life. In humans, spontaneous holes or defects in the IEL have been reported to exist in the iliac, femoral, splenic, and other large muscular arteries (23), and the coronary arteries (25). These defects are morphologically similar to the RIEL in the rat, both in histological section and en face preparations (17). Furthermore, IEL defects have been anticipated by various authors as initiator lesions of atherosclerosis in humans (9, 20) and reported to be so in ApoE-KO mice (16). Although it appears to be primarily a genetically determined characteristic, RIEL formation in the BN AA can be increased by the administration during the period of rapid growth of the lysyl oxidase inhibitors, β-aminopropionitrile (BAPN) and semicarbazide, and BAPN can even induce their formation before they appear spontaneously (7, 22). This observation led us to suspect the implication of a gene involved in elastin cross-linking. Previously, in a cross between the inbred strains BN and LOU, we identified a significant quantitative trait locus (QTL) for RIEL on chromosome 5 and a suggestive QTL on chromosome 10 (19). Proof of the implication of the chromosome 5 QTL was provided by producing congenic rats in which the chromosome 5 locus was transferred from the BN rat onto the genetic background of the reference LOU strain, but no physiologically relevant genes were known at the time to be located within this QTL.

In the present study, large cohorts of subcongenic lines were generated to further refine the chromosome 5 locus in order to isolate and prioritize the genes that are present. However, inadequate recombination events in the critical chromosome 5 region in the subcongenic lines prevented us from refining the QTL beyond 5.2 Mb with this method. We thus turned to the use of an outbred heterogeneous stock (HS), a robust genetic population for high-resolution mapping described in our previous study (2). HS were developed by the National Institutes of Health (NIH) in the 1980s and descended from eight known inbred progenitor strains, which included the BN: BN/SsN, MR/N, BUF/N, M520/N, WN/N, ACI/N, WKY/N, and F344/N (13) and were sustained by rotational breeding for >50 generations as described for mice (34, 37). Thus HS symbolizes a unique genetically random mosaic of founders that offers a high number of recombination events that have accumulated over many generations. In a mouse HS, the average recombination distance was reported to be ∼2 cM (37). We thus used a large cohort of outbred HS rats to further narrow down the QTL and reached a critical linkage interval of 1.4 Mb. Subsequent use of deep RNA-seq in the AA prioritized a protein coding gene and a putative regulatory noncoding transcript in this minimal region for RIEL phenotype.

## MATERIALS AND METHODS

### 

#### Animal breeding and genotyping.

Inbred BN/Orl RJ rats were from Elevage Janvier (Le Genest St Isle, France) and inbred LOU/M rats from our own breeding stock. Congenics LOU.BN(*D5Rat59-D5Rat133*) were generated as described previously (19) by transferring BN alleles of chr.5 QTL onto a LOU background. Here we produced a large population of recombinants by backcrossing the congenic LOU.BN(*D5Rat59-D5Rat133*) (50 Mb) to the resistant LOU/M followed by microsatellite and single nucleotide polymorphism (SNP) genotyping. Recombinants of interest were then bred to obtain homozygous individuals that were then bred to form six subcongenic lines. In this way, a large cohort of rats (∼1,000) of various genotypes was produced in several consecutive generations. All rats were kept in standard conditions with chow and drinking water ad libitum until the time of RIEL phenotyping. For genotyping, the tail tip was taken from each rat at around 5 wk of age and kept at −20°C until use. Genotyping was performed as described previously (19). NIH-HS rats originated from the colony established in the 1980s in the NIH (13). We phenotyped 1,002 HS rats, euthanized in four different batches at the Autonomous University of Barcelona, for RIEL. A subgroup of 559 of these rats was genotyped at 265,551 SNPs by microarray. This study was carried out within the framework of a large European collaborative study by the EURATRANS consortium (2).

#### RIEL phenotyping and quantification.

For RIEL phenotyping in subcongenics, male rats were killed at 20–40 wk and females at 30–50 wk of age. All rats were anesthetized with pentobarbital sodium (50 mg/kg), and their body weights and nose-rump lengths were recorded. Liver samples were snap-frozen in liquid nitrogen and stored at −80°C for genotyping if necessary. The AA and arteries distal to it were then perfusion-fixed with buffered formalin via an aortic catheter inserted at the level of the diaphragm (19). The AA and proximal 10 mm of IAs were then dissected out carefully and stored in buffered formalin until used. RIEL phenotyping was performed on en face preparations of AA and the attached IAs, by the method already described (19). For HS rats, adult males and females were killed between 15 and 21 wk of age by exsanguination under isoflurane anesthesia. The AA and proximal left IA (LIA) were rapidly dissected out, rinsed in saline, and fixed by immersion in formalin. RIEL were quantified, as for subcongenics, on en face preparations (2). For all rats, RIEL were quantified under the light microscope using ×4 objective (final magnification ×40). The distinction between true RIEL occurring spontaneously in vivo and mechanically induced artifacts occurring during experimental manipulation ex vivo was made under a higher magnification (×10 objective) to check the presence of endothelium or adhering platelets over the RIEL sites.

#### Treatment of congenic rats by BAPN.

The monofumarate salt of BAPN (Sigma), a lysyl oxidase inhibitor, was dissolved in physiological saline prior to injection, at a concentration of 0.091 mg/ml. Male subcongenic rats were treated from the age of 5 wk by a daily intraperitoneal injection of 0.2 ml per 100 g body weight of BAPN solution, corresponding to a dose of 100 mg/kg/day of BAPN free base. This dose has been used in previous studies (22, 28) and has been shown to affect arterial RIEL without having any general toxic effects. The treatment was stopped at 12 wk of age and rats were phenotyped at 14–16 wk of age.

All animal protocols for congenics were approved by the local Animal Ethics Committee of Inserm U698/Paris 7 University and carried out under authorization no. 75-825 of the Direction Departementale des Services Véterinaires de Paris, France. Animal protocols of HS were approved by the Autonomous University of Barcelona ethics committee (permit CEEAH 697).

#### RNA isolation and qRT-PCR analysis.

For RNA isolation, snap-frozen AA tissue from 5- to 6-wk-old male rats was resolved in Trizol reagent (Invitrogen) and immediately homogenized with an Ultra-Turrax homogenizer (IKA-T10 basic) without pooling different samples and subsequently treated with Turbo DNase (Ambion) according to the manufacturer's recommendations. We reverse-transcribed 10 μg RNA and converted it into double-stranded cDNA with the SuperScript II cDNA synthesis kit (Invitrogen). qRT-PCR was performed with TaqMan PCR master mix or SYBR Green PCR master mix (Applied Biosystems) on an ABI Prism 7900 sequence detector. For qRT-PCR, predesigned TaqMan gene expression assays used for candidate genes were protease inhibitor 15 (Pi15) (Rn01442644_g1, Rn01442648_m1), Crispld1 (Rno1442860_m1), GDAP1 (Rno1749151_m1), and Jph1 (Rn01448853_m1). The housekeeping β-actin (ACTB) gene was used as endogenous control (ABI). Primer sequences used for SYBR Green PCR for Pi15-exon2a and POLR2a were as follows: forward (F) 5′GCCATTCTTGATTACCATAA3′, reverse (R) 5′CAAGATGGCATAATGACTGTT3′; and F 5′TCGTATCCGCATCATGAACAG3′, R 5′GCACCGCAGGAAAACATCA3′.

#### Orientation-specific RT-PCR.

For strand-specific cDNA synthesis, we used 1 μg RNA of each BN, congenic, and LOU, including 2.5 μM oligonucleotide primers designed to detect the sense or anti-sense strands of putative aortic long intergenic noncoding RNA (lincRNA). Primer sequences of sense and anti-sense strands are 5′ATATTATGCGAAAATGACTAT3′ and 5′TTTAGCCTGCAAGATATACTT3′. The reverse-transcriptase reaction was performed with Superscript III (Invitrogen) according to the protocol provided with the kit. To verify the genomic DNA contamination, we performed a control reaction by omitting reverse transcriptase. Primer sequences used for strand-specific positive and negative strand RT-PCR were F 5′GGCTTGGTCTTATAACTTCC3′, R 5′TTTAGCCTGCAAGATATACTT3′; F 5′GGCTTGGTCTTATAACTTCC3′, R 5′ATATTATGCGAAAATGACTAT3′.

#### Sequencing reactions for candidate genes.

Candidate genes identified by expression analysis or located within the refined QTL were sequenced. Primers were designed with primer design program Oligo 6.0 and were synthesized by Biotez (Germany). Primer sequences used for nonsynonymous SNPs were Crispld1 F 5′AGATTGAAGGCTAATATGTGC3′, R 5′TCTCTATGTAGCTTTGGCTGT3′; and Gdap1 F 5′CGCATCTACGGTGTGA3′, R 5′ACTTCTCAGGGACAGATTAAC3′.

Genomic PCR was carried out using Taq DNA polymerase (Invitek). The amplified product was run on 2% agarose gel, after purification with nucleoSpin (Macherey-NAGEL), DNA sequencing was performed using BigDye Terminator V1.1 (Applied Biosystems) and sequenced on an ABI 3730 Sequencer (Applied Biosystems) according to the manufacturer's instructions. Sequences were aligned and analyzed using SeqMan from DNASTAR 5.10.

#### Mapping RIEL QTL in HS.

The total number of RIEL in AA and LIA were mapped by both a mixed-model method and a resampling method to account for relatedness in the HS and control the false discovery rate (FDR). The significance and posterior probability thresholds corresponding to an FDR of 10% were calculated by simulations as described previously (2). While the position of the QTL reported in Ref. 2 was an arbitrary 4 Mb interval centered on the interval most frequently detected by the resampling method, its location was investigated here by the mixed-model method and by simulation: in each simulation, a QTL of the same effect size as that of the real QTL was simulated in the vicinity of the position where the QTL was detected. The simulated phenotype was then mapped, and the position reaching the greatest −log *P* was recorded. That position was compared with the position where the QTL was simulated to obtain a distance D reflecting the imprecision in detection. The 90% confidence interval was defined as twice the 90th percentile of the distribution of D.

#### RNA-seq library preparation and data analysis.

Poly(A)+ RNA of AA tissue extracted from three BN, three congenic, and four LOU male rats, aged 5–6 wk, was sequenced on the Illumina HiSeq 2000 platform using TruSeq library preparation and 2 × 100 bp paired-end sequencing chemistry. Reads were mapped against the rn4 reference genome with tophat2 (35) and supplying transcript information as annotated by the Ensembl database (10) to aid the mapping process. Isoform locations were predicted in one BN, one congenic, and one LOU rat with the cufflinks suite and combined with the Ensembl annotation (36). Differential expression levels of novel and known transcripts between rat genotypes in the linkage region were detected with cuffdiff of the cufflinks suite.

#### Evaluation of longitudinal strain of AA and TA in situ.

A total of 48 rats (male and female BN, congenics, and subcongenics of lines A and B and LOU of different ages from 3 to 25 wk) were used for this part of the study. With the rats under deep pentobarbital anesthesia and after thoracotomy and laparotomy, the aorta was exposed from the arch down to the iliac bifurcation. Using the aortic arch, the superior mesenteric artery, the left renal artery, and the bifurcation as reference points, we employed fine calipers to measure the length of the AA and of the TA in situ, under a dissecting microscope when necessary. The two aortic segments were then excised, put into physiological saline at room temperature, allowed to shorten for ∼1 min, and then measured again. The difference between and the length in situ (l) and the length ex vivo (lo) divided by the length ex vivo [ε = (l − lo)/lo] permitted evaluation of the longitudinal strain (ε) to which each aortic segment is submitted in vivo.

#### Statistical analyses.

All results are presented as means ± SD, and expression studies were performed in triplicate or quadruplicate unless otherwise stated. Statistical analyses were performed using *t*-tests, the Kruskal-Wallis test followed by the nonparametric Mann-Whitney *U*-test, a one-factor ANOVA followed by the Tukey-Kramer test, a two-factor ANOVA, and the Spearman correlation test, where appropriate.

## RESULTS

### 

#### Dissection of the RIEL congenic region to within 5.2 Mb.

**Fig. 1.**
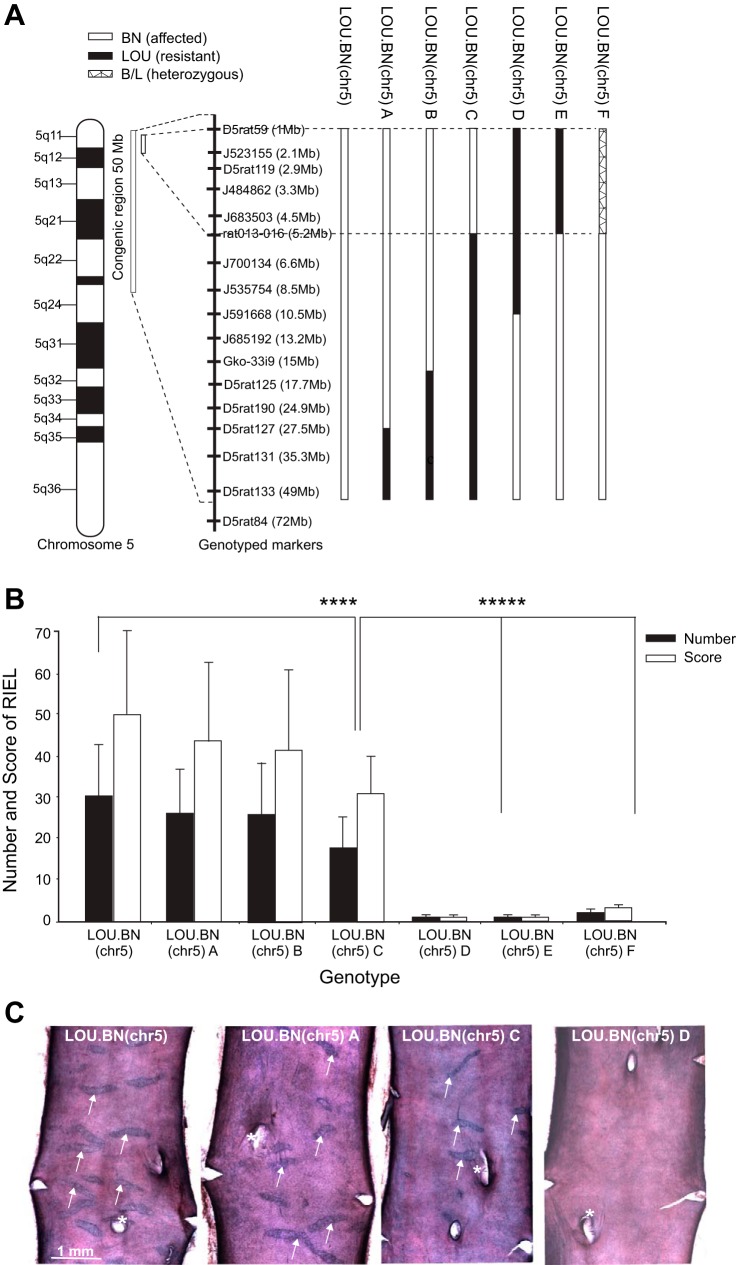
Schematic representation of subcongenic lines and rupture of internal elastic lamina (RIEL) phenotype. *A*: subcongenic lines developed from congenics LOU.BN(D5rat59-D5rat133). LOU.BN(chr5) represents the longest congenic segment (50 Mb). In LOU.BN(chr5) A and B the homozygous BN segment is refined to 27 and 15 Mb, respectively. LOU.BN(chr5) C possesses the minimal refined (5.2 Mb) BN segment with homozygous LOU alleles further down to 50 Mb; LOU.BN(chr5) D and E are the control LOU genotypes (10 and 5.2 Mb) with homozygous BN alleles further down; and LOU.BN(chr5) F represents a 5.2 Mb heterozygous segment. The scale, *center left*, indicates the position in Mb. Polymorphic microsatellites and SNPs were used to genotype the subcongenics and their physical (bp) position is given in parentheses. The refined BN segment is represented by dashed line. *B*: RIEL phenotype (number and score) of males of the above subcongenic lines, aged 20–40 wk. LOU.BN(chr5) congenics (50 Mb) exhibit the highest RIEL numbers and scores, while subcongenics LOU.BN(chr5) A and B (27 and 15 Mb) present a slightly less severe RIEL phenotype. The minimal subcongenics LOU.BN(chr5) C, carrying BN alleles at the 5.2 Mb proximal region, exhibit significantly higher RIEL numbers and scores than LOU.BN(chr5) D, E, and F, minimal LOU and heterozygous congenics; *n* = 89, 36, 40, 66, 38, 25, and 16 per genotype group. Means ± SD; ****P < 0.0001, *****P < 0.00002. *C*: examples of en face preparations of male AA showing RIEL (arrows) in congenic (50 Mb) and subcongenic lines A and C, and their absence in line D. *Origin of a lumbar artery on the dorsal side of the aorta.

The previously generated LOU.BN.chr5(*D5RAT59-D5RAT133*) congenics exhibited a 50 Mb BN interval on a LOU background (19). In the present study, a new panel of subcongenic lines, containing smaller segments spanning different sections of the LOU.BN(chr5) 50 Mb segment, were generated from the original congenics (Fig. 1*A*). In rats of all subcongenic lines, we recorded the total number of RIEL in the AA and IAs and also allotted a score, taking into account the size and severity of the ruptures, as previously described (19).

The total RIEL number and scores of congenics and subcongenic lines are shown in Fig. 1*B*, and examples of en face preparations of AA are shown in Fig. 1*C*. There was, as previously reported (19), a high correlation between RIEL numbers and scores (*P* < 0.0001), and so the two parameters are interchangeable. Phenotype to genotype correlation showed a genotype-dependent effect in all lines. The LOU.BN(chr5) (50 Mb) congenics and the LOU.BN(chr5) subcongenics A and B, containing homozygous BN segments from 0–27 and 0–15 Mb, respectively, showed high to moderate RIEL numbers and scores, while subcongenic LOU.BN(chr5) C, possessing the minimal BN introgressed region (0–5.2 Mb), displayed significantly greater RIEL numbers and scores relative to two other lines LOU.BN(chr5) D and E with homozygous LOU proximal segments [0–10 Mb (D) and 0–5.2 Mb (E)]. These two subcongenic lines (D and E) present negligible RIEL even though they carry BN alleles down to 45 Mb. A very weak phenotype was observed in LOU.BN(chr5) F, where the proximal segment (0–5.2 Mb) was heterozygous (BN/LOU). The minimal subcongenic phenotype revealed that gene(s) underlying the RIEL QTL are located within the proximal 5.2 Mb of chromosome 5 (between D5RAT59 and rat013–016). However, some modifying elements may be present within the distal 45 Mb region as this region appears to have a small effect on the RIEL phenotype.

In addition to BN×LOU, the aortic RIEL chromosome 5 locus was previously detected in another cross, BN×GH (14), and more recently BN.GK(chr.5) congenics were produced by transferring GK alleles from the chr.5 locus onto a BN background (D. Gauguier, unpublished data). As the GK is resistant to RIEL formation, aortic RIEL were reduced when the QTL region was composed of GK alleles. In these BN.GK.chr.5 congenics, the aortic RIEL locus was narrowed down to the proximal 15 Mb (D. Gauguier, unpublished data), including the proximal 5.2 Mb segment determined by the LOU.BN(chr5) subcongenics.

#### Sex difference and effect of age on RIEL.

**Fig. 2.**
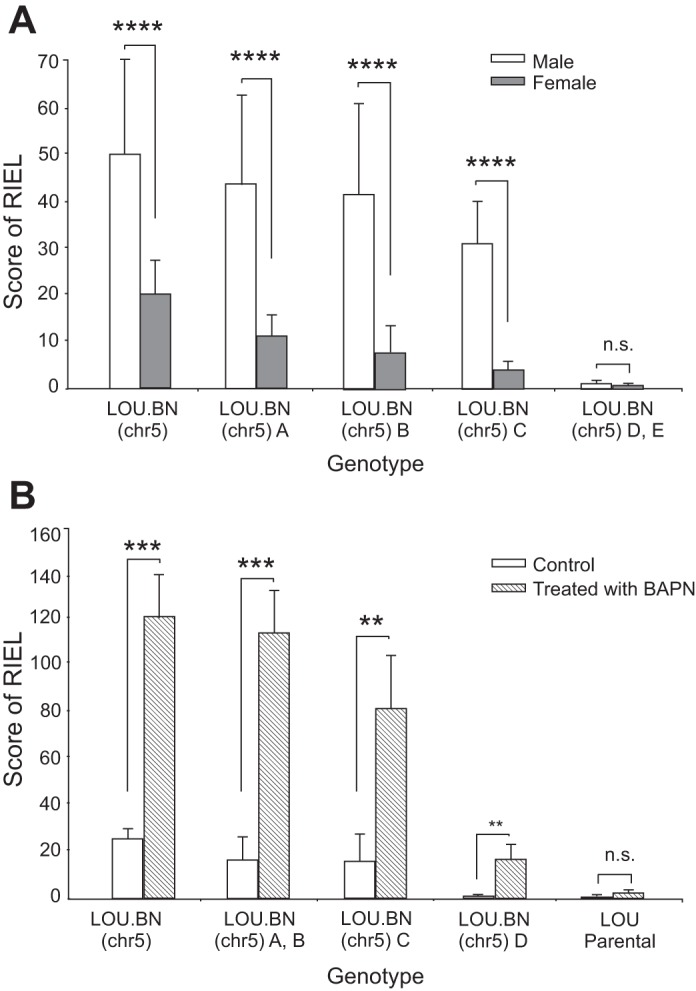
*A*: sex difference in congenic and subcongenic lines. A significant sex difference was observed for the RIEL score; *n* = 89, 36, 40, 66, 63 per genotype group for males, aged 20–40 wk, and 32, 28, 24, 29, 25 for females, aged 30–50 wk. Means ± SD. ****P <0.0001; n.s., nonsignificant. *B*: β-aminopropionitrile (BAPN)-treated congenic and subcongenic lines (aged 14–16 wk) exhibited significantly higher RIEL scores compared with untreated lines of similar genotype or with parental LOU rats. Interestingly, significant scores for RIEL were observed in the LOU.BN(chr5) D subcongenics after BAPN treatment; *n* = 8, 8, 4, 14, 6 per genotype group. Means ± SD; ***P < 0.001, **P < 0.02.

A significant sex difference in RIEL phenotype was observed in the subcongenic lines. Male subcongenics were more severely affected than females for an identical genotype (*P* < 0.0001), (Fig. 2*A*). Indeed, male congenic and subcongenic LOU.BN(chr5) A, B, and C carrying BN alleles for 50, 27, 15, and 5.2 Mb, respectively, exhibited greater RIEL scores than females with the same genotype, while LOU.BN(chr5) D (10.5 Mb) and E (5.2 Mb) males and females showed negligible RIEL [quantified data of LOU.BN(chr5) lines D and E were combined in Fig. 2*A* as both showed negligible RIEL scores in males and females].

In addition to the sex difference, in a preliminary study, male subcongenic lines were also studied at different ages (20 and 40 wk), and no significant difference was detected within any subcongenic line (data not shown), suggesting that, within the age range studied, genotype has a greater influence than age on RIEL formation. For this reason, in Figs. 1*B* and 2*A* males aged 20–40 wk are grouped, as are females aged 30–50 wk in Fig. 2*A*.

#### Effect of BAPN on RIEL.

Male subcongenic rats of different lines treated with BAPN, aged 14–16 wk, exhibited significantly greater RIEL scores compared with untreated rats of the same line (*P* < 0.001) (Fig. 2*B*). Indeed, the LOU.BN(chr5) (50 Mb) congenics reached scores comparable to the parental BN rat (19). Histologically, these ruptures were very similar to those that occurred spontaneously. The LOU.BN(chr5) D subcongenics, which had negligible RIEL when untreated, did show a small but significant increase in RIEL (*P* < 0.02), again suggesting that the chromosomal 5 segment from 10 to 50 Mb can have a modifying effect on the RIEL phenotype.

#### Exploring rat HS for fine-mapping RIEL QTL.

**Fig. 3.**
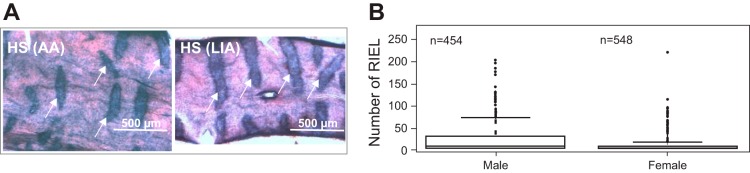
Phenotyping of heterogeneous stock (HS) abdominal aorta (AA) and left iliac artery (LIA) for RIEL. *A*: en face preparations of male AA and LIA. RIEL appear as dark blue transverse bands (arrows). *B*: box plot comparing RIEL numbers in HS rats of both sexes. Larger numbers of lesions were observed in males than in females. The box contains the middle 50% of the data, and the line within the box shows the median. The line above the box represents the 90th percentile, and the points above this, the values >90th percentile. As there are so many rats with 0 values, the lower 25th percentile coincides with the box bottom (*n* = 454 HS males and 548 HS females).

In contrast to the congenic lines, for which we could not refine the QTL beyond 5.2 Mb, the outbred HS rat offers a unique genetic resource that facilitates the rapid fine-mapping of QTL to small genomic regions (26, 37). We analyzed the genetic constitution of an experimental population of outbred HS for high-resolution mapping. The fact that BN was among the HS founder strains incited us to perform this study, as it meant that alleles associated with aortic and iliac RIEL should segregate in the HS colony. The AA and LIA of 454 male and 548 female rats were dissected and phenotyped for RIEL, as described in our previous report (2). Phenotyping was performed as for subcongenics, with minor modifications related to the difference in dissection and the lack of fixation in situ of the arterial segment (Fig. 3*A*). Phenotyping results demonstrated that the HS population as a whole exhibited a high variation in aortic and LIA RIEL. Moreover, consistent with the sex difference for RIEL observed in the subcongenic lines, significantly more ruptures were seen in male HS compared with females (Fig. 3*B*). In the total HS cohort of 1,002 rats, over one-third (401) were completely unaffected by RIEL, and relatively few (60) of them were moderately to severely affected (scores ranging from 60 to 222, including 45 males and 15 females). For reference, the BN rat had a mean score of 118 (males) and 65 (females) (19).

#### Mapping of the main genetic factor contributing to aortic and iliac RIEL to a 1.4 Mb-wide chromosomal region.

**Fig. 4.**
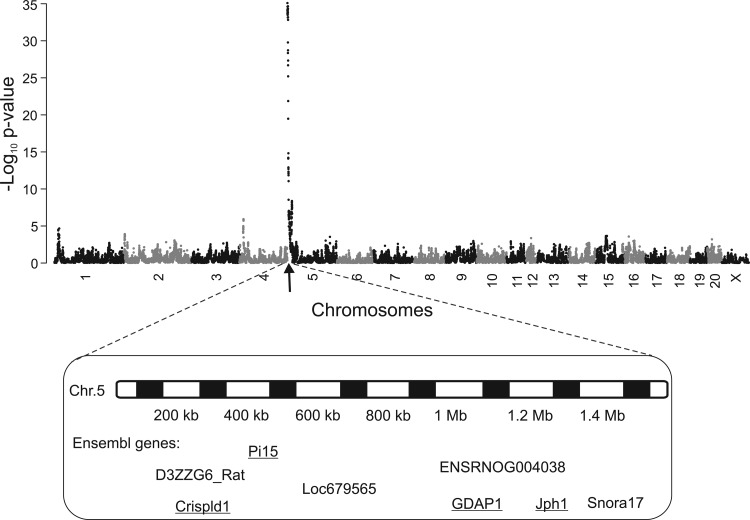
Association mapping of RIEL numbers in AA and LIA in HS rats. The *x*-axis indicates the chromosomal location, and the *y*-axis gives the negative log_10_ of the *P* value of the test of association. The genes shown lie in the 90% confidence interval of the QTL (1 bp to 1.4 Mb). Underlined genes are protein-coding, and others are hypothetical proteins.

RIEL in AA and LIA were mapped to the genome in a subset of 559 HS rats (chosen arbitrarily with respect to the RIEL phenotype) (2). We detected a major locus for AA and LIA score on chromosome 5 using resample model averaging (RMIP > 0.9) and mixed modeling (log *P* > 35), (Fig. 4). The 90% confidence interval for this locus was determined by simulations: the genetic factor(s) contributing to variation in aortic RIEL have a 90% chance of lying within the first 1.4 Mb of chromosome 5. The 1.4 Mb QTL encompasses only four protein coding genes (underlined) (rn4), shown in Fig. 4. Using the sequences of the HS founder strains, we determined that there were two nonsynonymous coding SNPs in this interval: one (T269I) in the cysteine-rich secretory protein LCCL domain containing 1 (Crispld1) and one (G162S) in ganglioside-induced differentiation-associated-protein 1 (Gdap1). However, since these amino acid changes were predicted to have benign effects on protein structure by PolyPhen (http://genetics.bwh.harvard.edu/pph/) and Crispld1 and Gdap1 were very weakly expressed in the aorta, these amino acid changes were considered unlikely to be responsible for the QTL.

#### RNA-seq analysis revealed a protein coding gene and a noncoding transcript in the critical 1.4 Mb linkage interval.

**Fig. 5.**
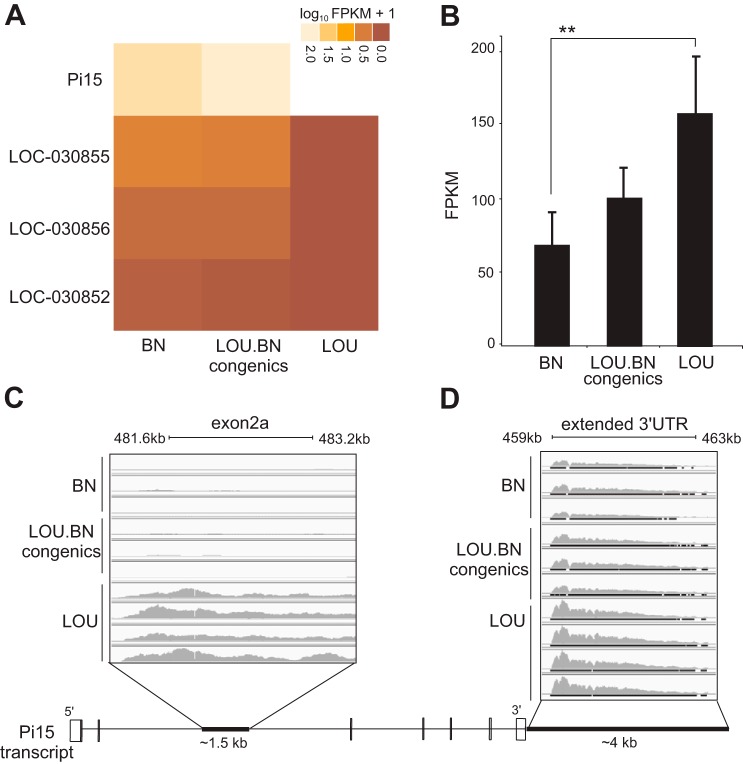
RNA-seq analysis in aorta. *A*: a heat map showing the significant gene expression in the 1.4 Mb linkage interval between the different groups. Light shading represents overexpression, and dark underexpression. LOC030855 and LOC030856 are putative lincRNAs, while LOC030852 is a predicted pseudogene. *B*: genome-wide significant differential expression for Pi15 between BN and LOU. FPKM, fragments per kilobase of exon per million fragments mapped. **q value < 0.02. *C*: black bar (*bottom*) reveals a 1.5 kb alternative exon 2a sequence overlap within intron 2 at position (481.6–483.2 kb). The sequence was highly expressed in LOU compared with BN and congenics. *D*: an extended 3′-untranslated region (UTR) signal captured for protease inhibitor 15 (Pi15) at position 459–463 kb beyond the annotated RefSeq of 3′-end (shown by black bar). *Bottom*: a schematic diagram of the Pi15 transcript.

To identify strong positional candidates for RIEL, we carried out a comprehensive analysis of transcript expression using RNA-seq in the abdominal aortic tissue of three biological replicates of BN and congenics and four replicates of LOU. In total, 120 million properly paired reads were aligned to the reference genome (rn4). We used a strict selection criterion for the candidate genes that showed significant consistent differential expression between BN, congenics (RIEL-affected) vs. LOU (control) genotypes. Since BN and congenics carry the same alleles at the chromosome 5 locus, their concordant expression was considered as a necessary criterion for candidates. We thus identified three differentially expressed transcripts on a genome wide-scale (FDR < 0.05) in the linkage region (1.4 Mb): a gene coding for the protein Pi15 (ENSRNOG00000017686), a fragmented putative lincRNA (LOC_030855, LOC_030856), and an annotated pseudogene (LOC_030852) (Fig. 5*A*). Indeed, the 1.4 Mb interval harbors four protein coding genes (Fig. 4), but only Pi15 passed the test regime of significance and sustained differential expression across strains (Fig. 5*B*). In addition, the RNA-seq analysis revealed a putative alternative exon 2a of ∼1.5 kb overlapping with intron 2 of Pi15 and further suggested a longer 3′-untranslated region (UTR) beyond the RefSeq annotated 3′-end of ∼4 kb (Fig. 5, *C* and *D*).

**Fig. 6.**
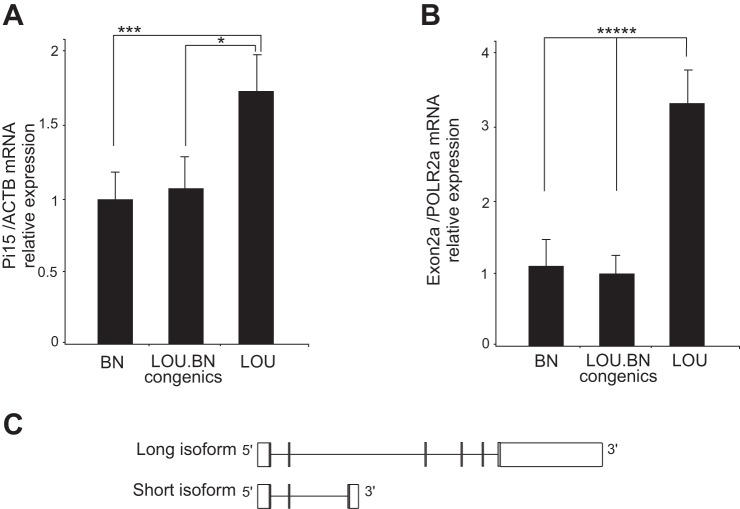
Validation of RNA-seq analysis. *A*: qRT-PCR showed significant differential expression for Pi15 in BN and congenics compared with LOU; *n* = 8, 8, 9 per each group. Means ± SD. *P < 0.02, ***P < 0.003. *B*: RT-PCR of Pi15 exon 2a showed an increased expression of the alternative exon in the LOU compared with BN and congenics; *n* = 5 per each group. Means ± SD. *****P < 0.00007. *C*: schematic representation of short and long isoforms of Pi15. Shaded bars show exons, and open bars represent UTRs.

The expression level of Pi15 was assessed by qRT-PCR (Fig. 6*A*). Consistent with the result of RNA-seq, the relative Pi15 mRNA levels in the aorta of the LOU were significantly higher compared with the BN and congenics. The existence of an alternative exon 2a was confirmed by RT-PCR. RT-PCR primers, spanning from exon 2 and exon 2a, indicate the presence and consistent differential expression of an alternative exon between the strains (Fig. 6*B*). The occurrence of the alternative exon leads to the short noncoding isoform of Pi15 that carries exon 1 and 2 and an alternative exon 2a. The extended 3′-UTR sequence in rat has been annotated in human and mouse and shows conservation. The long and short Pi15 isoforms are shown schematically in Fig. 6*C*. Overall, the mRNA levels of both Pi15 isoforms were significantly decreased in the BN and subcongenics compared with LOU rats (*P* < 0.003 and *P* < 0.00007) and thus may display decreased Pi15 mRNA levels in the aorta. Pi15, otherwise called trypsin inhibitor, is a secreted protein. Trypsin displays strong protease activity against extracellular matrix proteins, such as elastin and fibronectin (24). Due to the functional relevance of Pi15 with aortic IEL ruptures and its significantly decreased expression in the affected rats, one may suggest that Pi15 could play a defensive role against proteolytic damage to elastic tissue.

**Fig. 7.**
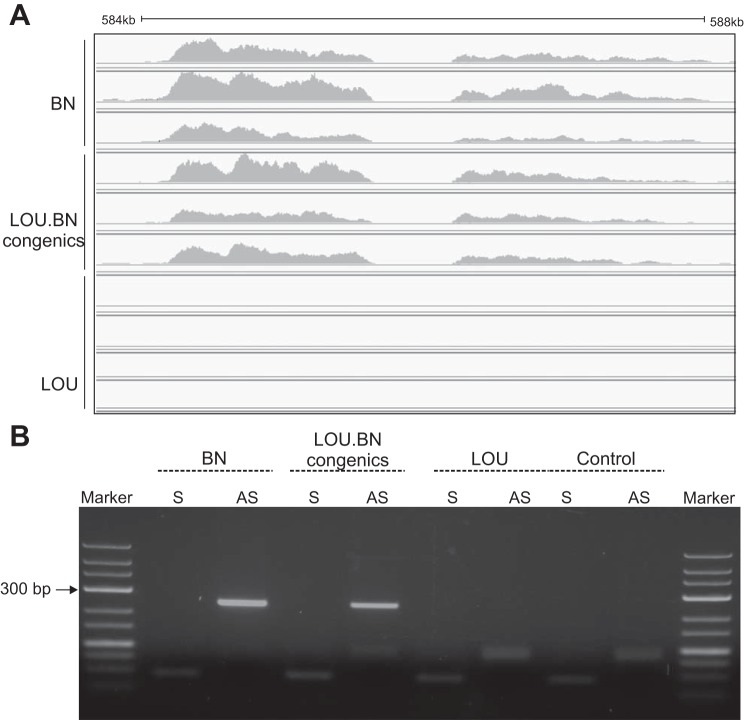
RNA-Seq detected a putative long intergenic noncoding RNA (lincRNA). *A*: a high expression of lincRNA was detected at position (584,500–588,000 bp) in BN and congenics but not in LOU. They are located ∼90 Kb upstream to Pi15. *B*: strand-specific RT-PCR demonstrates the putative lincRNA expression on the opposite strand in BN and congenics but not in LOU. The control reaction indicates the absence of genomic DNA contamination by omitting the reverse transcriptase. S, sense; AS, anti-sense.

In addition to the strong candidate Pi15, a putative lincRNA was captured upstream of Pi15 at position 584500–588000 bp within the intergenic region. Due to the sequencing gap in the reference genome (RGSV3.4 and http://www.ucsc.edu), the putative lincRNA mapped in two adjacent fragments (Fig. 7*A*). They were highly abundant in BN and congenics but not expressed in LOU. However, the lincRNA mapped reads (∼4 kb) possessed LINE-1 (L1) elements (http://www.repeatmasker.org) and were poorly conserved (http://www.ucsc.edu). Due to the presence of repeat elements (L1), only a partial unique sequence could be validated by orientation-specific RT-PCR, which demonstrated putative lincRNA expression on the opposite strand in the affected rats (BN and congenic) but not in the control (LOU) rat (Fig. 7*B*). It is known that many human lincRNAs are expressed in a more tissue-specific manner than protein coding genes and are located proximal to developmental regulators (6). With that in mind, we checked the putative lincRNA expression in other rat tissues but could not detect expression in the liver or left ventricle by RNA-seq in BN and SHR (unpublished data) or in the cell lines C6, PC12, and H9C2, derived from rat glioma, adrenal medulla, and cardiomyocyte cells, respectively. Thus our novel putative lincRNA exhibits tissue/cell-specific expression. Overall, the great abundance of putative lincRNAs with L1 in the affected rats (BN, congenics) and the downregulation of Pi15 in the same rats may indicate their function in the regulation of Pi15 expression.

#### Comparison of Pi15 regulatory sequences of BN, congenics, and LOU.

The sequence analysis of Pi15 identified many SNPs and insertion/deletions within the intron and regulatory region. A 2 bp indel (AGA/A–) found in the 3′-UTR between BN and LOU at position 43 bp relative to the translation stop site and repeat polymorphisms detected between strains were (TA)_21_/(TA)_23_ and (TG)_17_/(TG)_22_. This may suggest that repeat polymorphisms within the 3′-UTR may contribute to the regulation of Pi15 expression. However, this needs to be further validated.

The newly detected ∼4 kb 3′-UTR sequence alignment with orthologous regions of human and mouse showed several conserved regions (http://www.ucsc.edu). Further experiments are needed to study the effect of SNPs and in/dels in altering the gene expression within the strains.

#### TA vs. AA.

**Fig. 8.**
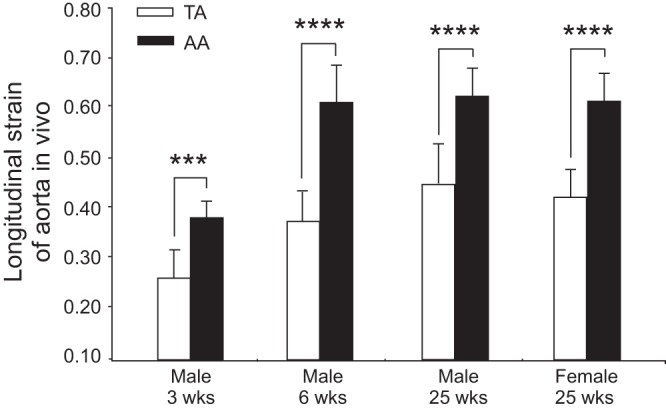
Evaluation of longitudinal strain of AA and thoracic aorta (TA). Males of 3, 6, and 25 wk of age and adult females show higher longitudinal strain in vivo in AA compared with TA; *n* = 5, 15, 13, and 10 per group. Means ± SD. ***P < 0.005, ****P < 0.00001.

In view of the fact that the TA never develops RIEL, even in the BN rat (7), it was of interest to investigate whether expression differences observed in the AA were also present in the TA. Such differences do exist although are less marked for Pi15 in the congenic TA (results not shown). This indicates that the high susceptibility of the AA to RIEL is probably rather due to some local influence. We thus tested the hypothesis that the AA undergoes more longitudinal strain during growth than the TA, by measuring the in situ length of the AA and TA and then the length of the same segments after excision. In all cases in male rats, at different ages and in the different strains, the longitudinal strain was significantly higher for AA than for TA (Fig. 8), and a similar result was found in adult female rats.

## DISCUSSION

Previous work identified a major locus for RIEL in AA and IA on chromosome 5. Subsequently, congenics were generated by transferring the BN QTL onto a LOU background (19). In the present study, we have produced a large number of subcongenics with shorter congenic segments to identify the gene (s) responsible for the aortic RIEL locus. We thus refined the locus down to 5.2 Mb by producing minimal subcongenics. Significant numbers of lesions were observed in subcongenic lines that present the BN alleles in the first 5.2 Mb, but lesions were completely absent when resistant LOU alleles occupied this position, even if the remaining 45 Mb was covered by BN alleles. The detailed genotype/phenotype analysis in subcongenic lines demonstrated genotype- and sex-dependent effects. For each genotype, male subcongenics were more severely affected by RIEL than females. This male-female difference in aortic RIEL is a constant trait in all rats studied: BN, backcross rats, and congenics. It was also observed that congenics with longer homozygous BN segments in the QTL region (down to 50 Mb) develop more lesions compared with those with shorter BN segments (down to 5–10 Mb), suggesting some modifying effect of the more distal congenic segment.

Moreover, BAPN-treated subcongenics exhibited significantly more lesions than age-matched untreated rats of the same genotype. This BAPN effect is in accord with previous reports in the Wistar (28) and the BN rat (7, 22). Lysyl oxidase, which is inhibited by BAPN, catalyzes the formation of cross-links in elastin and collagen fibers and plays a critical role in maintaining homeostasis of the elastic lamina (8). BAPN also induced a small but significant number of RIEL in minimal congenics where LOU alleles occupied the proximal 10 Mb and no RIEL were observed in untreated rats. This also suggests that the more distal chromosomal segment (between 10 and 50 Mb) can have a modifying effect on IEL rupture. However, none of the lysyl oxidase genes or other genes potentially affecting elastin cross-linking are present on chromosome 5. These results nevertheless illustrate the importance of gene-environment interactions (*P* < 0.0001, 2-factor ANOVA) in RIEL formation.

Previous work has identified the involvement in RIEL formation of a second QTL on chromosome 10 in both the BN×GH cross (14) and the BN×LOU cross (19). Work on BN (GK) congenics has also confirmed that this locus can play a significant role in the RIEL phenotype (D. Gauguier, unpublished observations). However, in the present work on congenics, this locus is not operating as it is occupied by LOU alleles. This fact probably explains, at least in part, the lower levels of RIEL in our congenics compared with the parental BN rat (19). It is also noteworthy that this chr10 locus was not detected in our HS study.

During the present study, where a very large number of rats were phenotyped, it was noted in occasional congenics and subcongenics of lines A, B, and C that, at the site of some RIEL, the aortic media was hollowed out and appeared to show thinning and local dilatation, evoking an aneurysmal lesion. This type of microaneurysmal lesion was also observed in two younger BAPN-treated congenic males [one congenic (50 Mb) and one subcongenic, line A] and also in some HS rats. In all cases, this type of dilating lesion was located on the dorsal side of the aortic wall, proximal to the origin of a lumbar artery, suggesting that RIEL in a particular anatomical location could represent starting points for aneurysm development. Such observations suggest that causal gene(s) underlying the aortic RIEL may be involved in aneurysm initiation. It is nevertheless probable that the adventitia, which constitutes a mechanically resistant type-1 collagen sheath surrounding the media, would have to be weakened to permit full-blown aneurysm dilation.

However, in contrast to one previous report (18), in the parental BN rats used in this study, the spontaneous RIEL in the AA and IAs, even when very large, never evolved toward microaneurysm formation. This was true for very old BN rats or for younger ones or in the presence of hypertension or after lysyl oxidase inhibition (27). This suggests that such an evolution can occur only on a genetic background other than pure BN and would explain why the BN has a normal life-span despite multiple aortic and iliac RIEL. Consistent with this, in a BN×GH cross, Jones et al. (15) also noted some “deep” lesions at sites of IEL rupture, not seen in BN.

In view of the numerous genes present in the 5.2 Mb interval defined by the subcongenic study, we then assessed the phenotypic variation in AA and LIA RIEL within an HS population of 1,002 rats and conducted fine-mapping. So far, in the rat only few studies have used HS for fine-mapping. The present work is the first time that the aortic RIEL trait has been measured in a large HS colony for QTL analysis as described briefly in our recent report (2). The fact that a large number of rats (∼⅓ of the HS population) exhibited very few to no detectable lesions in their arteries, while very few rats exhibited large numbers of lesions, provided promising conditions for understanding the genetic factors contributing to aortic RIEL and its severity. As observed in congenic and subcongenic rats, in HS, a high correlation (*P* < 0.0001) was found between RIEL numbers and scores, and a significant association was seen with sex as already observed in past studies in which males had more IEL lesions than females. This recalls the situation in humans where men are more susceptible to AA aneurysm formation, and indeed also to other forms of atherosclerosis, than women.

At the outset of this study, it appeared that only the BN genome could be responsible for aortic RIEL in the HS population. Indeed, of the eight founder strains, the BN is to date the only one known to present numerous aortic RIEL. We performed a limited phenotypic analysis of the ACI rat, another founder strain of the HS cohort, which revealed a mild phenotype (score 17 ± 7, males, *n* = 10). Thus the ACI background could also contribute to RIEL incidence in the HS population. Of the other HS founder strains, the F344 exhibits negligible aortic RIEL numbers (4), and the others have not been studied in this respect. WN/N and WKY/N, were derived from Wistar stock and so are unlikely to present the phenotype since the Wistar and closely related strains (SHR, Wistar Furth) do not develop aortic RIEL (M. Osborne-Pellegrin, unpublished observations), while MR/N, BUF, and M520 are unavailable from commercial sources and so their phenotype remains unknown. However, genotyping of the parental strains showed BN-like alleles in the chr5 locus for both ACI and MR/N, suggesting that this latter may also present the phenotype to some degree.

The genetic mapping in the HS identified and confirmed an extremely significant association of RIEL with chromosome 5 located within a small 90% confidence interval of 1.4 Mb. After that, RNA-seq study in the aorta revealed a genome-wide significant expression difference for a protein coding gene, Pi15, and a repetitive element-containing novel lincRNA within the minimal 1.4 Mb linkage region. Pi15 is also known as 25 kDa trypsin binding protein. It is a secreted protein and localized to the extracellular matrix (11). It is known that trypsin can degrade various extracellular matrix proteins and can activate metalloproteinases. It could thus be involved in the pathophysiology of vascular diseases. Trypsin also causes endothelium-dependent vasorelaxation in the rat aorta and other blood vessels (1). A previous study from our group demonstrated that latent elastinolytic activity of aortic endothelium (after trypsin activation) is greater in BN than in another strain without aortic RIEL (29). Moreover, Pi15 expression was previously detected at a precise developmental stage in the chicken during organogenesis (33), suggesting its role in development. Furthermore, Pi15 expression has been detected in the human atherosclerotic aortic wall (12) and in the hyperlipidemic mouse aorta (3).

In the present study, an alternative exon was detected for Pi15 that resulted in a short isoform. Both long and short isoforms were significantly downregulated in the affected rats. Such reduced Pi15 (trypsin inhibitor) expression may result in increased protease activity in the aorta consistent with a role for Pi15 in aortic RIEL. Our RNA-seq experiment has also detected a putative lincRNA expression upstream to Pi15. These elements encompass LINEs and are located on the opposite strand relative to Pi15. They are highly expressed and were validated only in the affected rats, a fact that leads to speculation on their role in the regulation of Pi15 expression. However, putative lincRNA showed a tissue-specific expression, and further experiments are needed to investigate its potential role in downstream Pi15 gene regulation.

We have shown that similar expression differences exist between the two strains also in the TA, suggesting that the high susceptibility of the AA to RIEL is probably rather due to some local influence. Our measurements of aortic length in situ and ex vivo show that in all cases, at different ages, and in the different rat strains, the AA was under more longitudinal strain in situ than the AT. This finding may explain, in part, the different susceptibilities of the two aortic segments to RIEL formation in the presence of genetic variations relating to this phenomenon. Indeed, experimental longitudinal traction and strain applied to arteries ex vivo induce transverse tears in the IEL (5) similar to the RIEL studied here. The fact that the same result concerning AA longitudinal strain was obtained in female rats does not explain the male-female difference in RIEL incidence. However, in view of the considerable sexual dimorphism existing in the rat, the growth in length of the aorta, presuming it is proportional to growth in body length, should be considerably less in females than in males (between 6 and 25 wk of age the nose-rump length increases by 0.448 in males and only by 0.331 in females). The reduced longitudinal growth in females may influence RIEL formation in the AA as already reported for the caudal artery after estrogen treatment (31).

Another possible explanation for the fact that only the AA is affected by RIEL may be the different embryological origins of the thoracic and abdominal aortic segments (21). Both experimental work in dogs and studies in humans have provided evidence that each arterial segment has its own intrinsic susceptibility to atherosclerosis development, which appears to be independent of its location (for review see Ref. 21).

The reasons for the excessive susceptibility of the human AA to aneurysm formation remain open to speculation. The previously proposed characteristics of the human AA, i.e., a reduced number of lamellar units and fewer vasa vasorum (38), together with lineage-dependent differences in smooth muscle cell properties (21) that may alter their response to pathological stimuli, probably play a role. Whether longitudinal strain is also a contributing factor will require further investigation.

In summary, we have integrated multiple approaches to identify genetic factors involved in aortic and iliac RIEL in different groups of rats: congenics and subcongenics derived from BN and LOU inbred strains and an HS population. We also observed, for the first time, that occasional RIEL in the large population of rats studied appeared to start to evolve toward aneurysmal-like lesions. Our data concerning the fine-mapping of a previously identified major chr5 QTL for RIEL, alongside expression analysis in the aorta, unveiled a critical 1.4 Mb linkage interval and identified a highly expressed protein coding gene, Pi15, and an upstream repetitive element-containing putative lincRNA within the 1.4 Mb region. Moreover, the high abundance of lincRNA in the affected rats (BN, congenic), coincident with the downregulation of Pi15, may suggest their function in the regulation of Pi15 expression. It is indeed physiologically conceivable that decreased aortic Pi15 expression, at some point in the development of the IEL, may cause structural fragility, leading to the formation of IEL rupture under hemodynamic and longitudinal tensile stress later in life, and in particular during the period of rapid growth associated with aortic elongation. Whether such a process is involved in a predisposition to human aneurysm formation remains to be elucidated.

## GRANTS

This work was funded by Inserm, the European Union's Seventh Framework Programme FP7/2007-2013 under Grant Agreement HEALTH-F4-2010-241504 (EURATRANS), and Wellcome Trust Grants WT089269 and WT083367/07.

## DISCLOSURES

No conflicts of interest, financial or otherwise, are declared by the author(s).

## AUTHOR CONTRIBUTIONS

Author contributions: S.F. and M.O.-P. performed experiments; S.F., S.S., A.B., O.H., H.S., and M.O.-P. analyzed data; S.F., D.G., N.H., and M.O.-P. interpreted results of experiments; S.F. and M.O.-P. drafted manuscript; S.F., N.H., and M.O.-P. edited and revised manuscript; N.H. conception and design of research.
